# Damages caused by hurricane Irma in the human-degraded mangroves of Saint Martin (Caribbean)

**DOI:** 10.1038/s41598-019-55393-3

**Published:** 2019-12-12

**Authors:** R. Walcker, C. Laplanche, M. Herteman, L. Lambs, F. Fromard

**Affiliations:** 1 EcoLab, Université de Toulouse, CNRS, Toulouse, France; 2 Nature & Developpement, Martinique, France

**Keywords:** Environmental health, Tropical ecology, Wetlands ecology

## Abstract

In early September 2017, Irma was the most powerful hurricane that struck the northern Caribbean over the last 100 years. In the 21^st^ century, the stronger types of tropical cyclones will likely increase in frequency due to the climate change and internal climate variability. Lessons to anticipate the response of mangroves to this intensification can be learned from this extreme event. Here, we analysed damages caused in mangrove forests of the Saint Martin Island. Mangroves of this island were previously degraded due to historic human pressures and recent over-urbanisation. Forest inventories and time series of very high resolution satellite images revealed that approximately 80% of the mangrove area was damaged by the hurricane. Results highlighted distinct rates of forest recovery. Early and rapid recoveries were largely observed in most study sites. However, some mangroves were still unable to recover fourteen months after the disturbance. The human-induced degradation of the ecosystem prior to the hurricane is hypothesised to be the main factor controlling the absence of forest recovery. We suggest that human-degraded mangroves will be weakened in the face of such extreme events. We advocate to preserve and restore mangroves in order to guarantee all the valuable ecosystem services they provided.

## Introduction

Between 1996 and 2016, approximately 6 000 km² of mangrove have been lost globally, mainly due to the development of aquaculture, agriculture, urbanisation and coastal infrastructures^1^. Many ecosystem services provided by mangroves have been affected, including nurseries that maintained biodiversity and productivity of coastal waters, natural defences against tsunamis, and sinks for the carbon sequestration^2–4^. A recent review of the published literature over the last six decades has suggested that tropical cyclones may have significantly contributed to mangrove loss^5^. Indeed, high energy winds combined together with destructive waves and storm surges have caused severe damages to mangrove habitats throughout the world^6–9^. Under natural conditions, mangroves are very resilient ecosystems and damages caused by tropical cyclones are rarely persistent^10,11^. However, we hypothesised here that combined together with multiple human-induced stressors – e.g. alteration of flow regimes, pollution, over-harvesting, etc. – tropical cyclones can result in persistent, potentially irreversible damages. Indeed, we suspect that the consequences of tropical cyclones in mangroves will be amplified by prior alteration of ecosystem productivity, nutrient cycling or hydrological circulation induced by human activities^5,12^.

**Figure 1:**
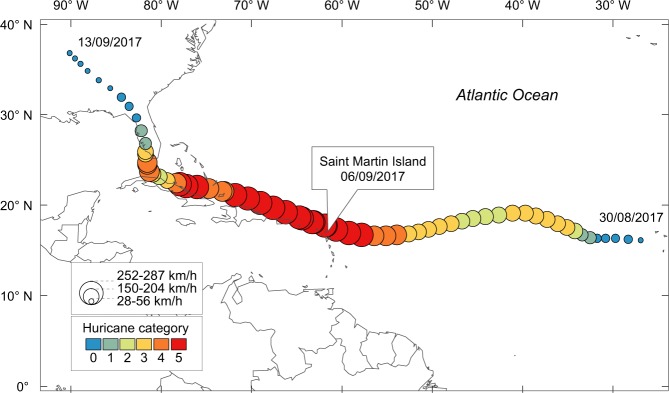
Hurricane Irma path in the North Atlantic. According to the Saffir-Simpson hurricane wind scale, Irma was rated as a category 5 hurricane when it made landfall on Saint Martin, on the 6th September 2017. Irma was the most powerful of the 19 hurricanes that struck the Saint Martin Island over the last century. Data source: NOAA, National Hurricane Center.

At global scale, tropical cyclones and associated extreme weather events will likely to increase due to the climate change and the internal modes of climate variability^13–16^. A better understanding of the impacts of tropical cyclones in the mangrove development is urgently needed. Here, we investigated damages caused by Irma, the most severe tropical cyclone that struck the Saint Martin Island (Lesser Antilles) over the last century. Saint Martin is situated approximately 300 km east of Puerto Rico and 200 km North-West of Guadeloupe (Fig. 1). Mangroves of Saint Martin are limited in space and are mostly degraded due to historic activities of salt extraction in evaporation ponds and to the recent over-urbanisation. Irma’s eyewall struck Saint Martin Island as a category 5 hurricane on the 5–6 September 2017. Winds exceeded 287 km/h and waves reached 8 m height (Fig. 1; Table S1). Time series of remote sensing observations (Table 1) coupled with ground observations (Table S2; Supplementary Dataset) were used to quantify damage, mortality and early signs of mangrove forest recovery.

**Table 1:** List of aerial and satellite images used in this study.

Image date	Time from Irma	Supplier	Sensor	Bands	Pixel size
2004	−13 yr.	IGN	Digital photo	R, G, B	~0.5 m*0.5 m
2011/02/26	− 6.6 yr.	MapMart	Worldview 2	R,G,B,NIR	~0.5 m*0.5 m
2013/01/02	−4.7 yr.	GEOSUD	Pléiades	R,G,B,NIR	~0.5 m*0.5 m
2017/02/25	−0.6 yr.	ISIS	Pléiades	R,G,B,NIR	~0.5 m*0.5 m
2017/09/10	+4 d.	GEOSUD	Pléiades	R,G,B,NIR	~0.5 m*0.5 m
2017/11/30	+0.2 yr.	ISIS	Pléiades	R,G,B,NIR	~0.5 m*0.5 m
2018/02/03	+0.4 yr.	GEOSUD	Pléiades	R,G,B,NIR	~0.5 m*0.5 m
2018/10/28	+1.2 yr.	GEOSUD	Pléiades	R,G,B,NIR	~0.5 m*0.5 m

## Results

### Results from field campaigns

#### Changes in tree health, size and stem density

**Figure 2:**
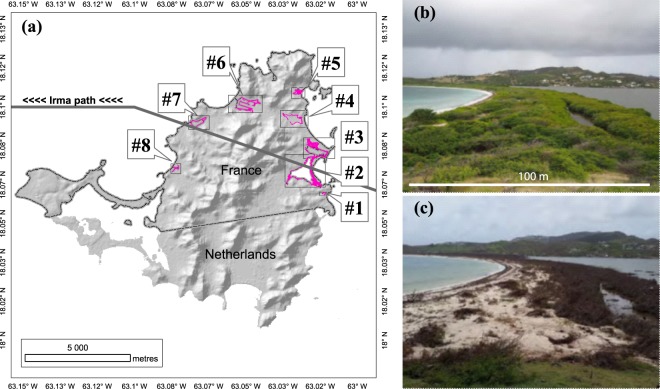
Impact of Irma on mangrove forest of *Etang aux Poissons* (site #2), eastern coast of Saint Martin Island. **(a)** Distribution of study sites in the Saint Martin Island. **(b)** Site #2 before Irma in 2017. Mangroves and associated species of this coastal barrier were in a healthy state. **(c)** Immediately after Irma in 2018, mangroves and associated species completely died (© Julien Chalifour).

**Figure 3:**
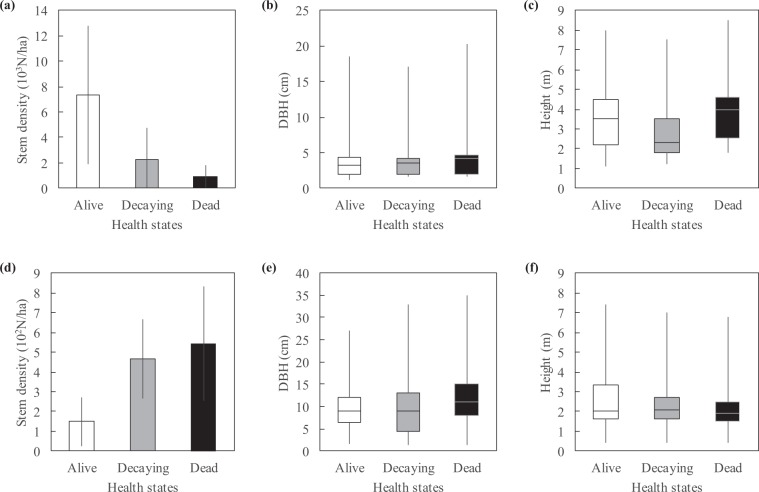
Stem density, trunk diameter and tree height as a function of tree health state (**a–c**) before Irma in 2011 and (**d–f**) after Irma in 2018. Before Irma, stands were dominated by living trees, while dead and decaying trees accounted for 7% and 26%, respectively. After Irma, dead and decaying trees accounted together for 90%. No significant relationships were found between tree health state and trunk diameter or tree height.

**Figure 4:**
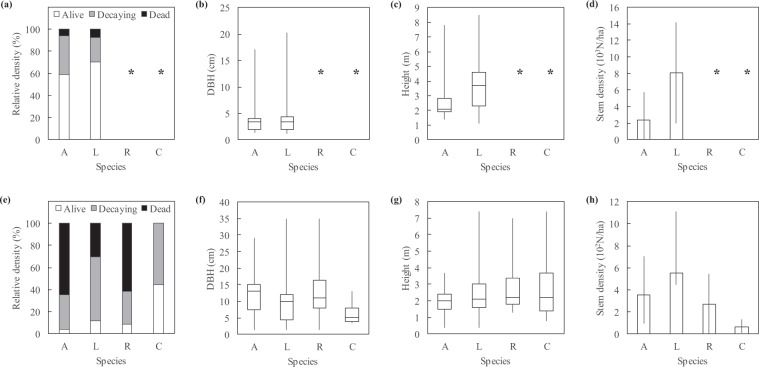
Relative density of tree health state, trunk diameter, tree height and stem density as a function of species **(a–d)** before Irma in 2011 and **(e–h)** after Irma in 2018. A, L, R and C denote *Avicennia germinans*, *Laguncularia racemosa*, *Rhizophora mangle* and *Conocarpus erectus*, respectively. *Corresponds to non-inventoried species.

**Figure 5:**
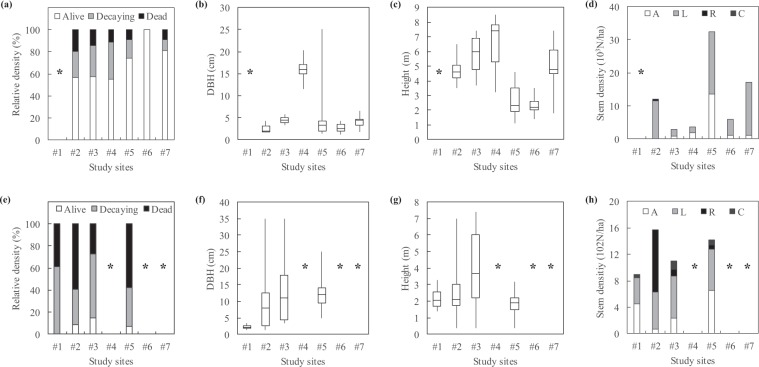
Relative density of tree health state, trunk diameter, tree height and stem density as a function of study sites **(a–c)** before Irma in 2011 and **(d–f)** after Irma in 2018. *Corresponds to non-inventoried sites.

Forest inventories carried out before and after Irma (June 2011 and April 2018) revealed changes in stand health and structure (Fig. 2). Trees significantly differed in health before and after Irma (ANOVA’s Date effect on Health; Table 2). Before Irma, the 266 inventoried trees were largely dominated by living trees (Table S2). Alive, decaying and dead trees accounted for 67.3%, 25.9% and 6.8%, respectively (Fig. 3a). After Irma, the 169 inventoried trees were largely dominated by dead and decaying trees (45.6% and 43.8%, respectively) whereas living trees accounted for only 10.7% (Fig. 3d). Stem densities were also highly different before and after Irma (Table 2), switching from thousands down to hundreds of stems per hectare (Figs. 3a,d, 4d,h, 5d,h). Means of trunk diameters and tree heights also differed before and after Irma (Table 2). Remaining trees were larger in diameter (Figs. 3b,e, 4b,f, 5b,f) and lower in height (Figs. 3c,f, 4c,g, 5c,g).

**Table 2:** P-values obtained from ANOVAs using forest inventory data aggregated at the plot level. Asterisks denote signification levels: ***is <0.01, **is <0.01, and *is <0.05.

Factor	Response variable			
	*Health*	*Density*	*DBH*	*Height*
*Date*	<0.0001***	<0.0001***	0.0005***	<0.0001***
*Species*	0.26	0.0009***	0.87	0.41
*Site*	0.02*	0.31	<0.0001***	<0.0001***
*Date-Site*	0.96	0.06	0.90	0.75
*Date-Species*	0.80	0.96	0.93	0.47

DBH, Height and Density were log-transformed to fulfil ANOVA requirements. P-values of the Shapiro-Wilk normality and Levene homoscedasticity tests were all >0.05, except Levene’s test for DBH (p-value = 0.0004). The Date-Site and Date-Species interaction factors evaluates if differences before and after Irma were site- and species-dependent.

#### Species-dependence of hurricane damages

The tree species slightly modulated the effect of Irma. Analyses showed that the effect of Irma on *A. germinans* and *L. racemosa* health, size (trunk diameter and tree height) and density was not species-dependent (ANOVA’s Date-Species effects; Table 2). However, after Irma when considering all 4 sampled species altogether, tree health state and species are found as significantly dependent (Fig. 4e; Fisher’s exact test, p-value < 0.0001). *C. erectus* was the species that better resisted to the hurricane with only 56% of decaying trees. Dead *L. racemosa* were found in lower proportions (31%) than those of *A. germinans* and *R. mangle* (65% and 62% respectively). Decaying *A. germinans* and *R. mangle* were found in same proportions (31% and 29%, respectively), as well as for living trees (4% and 9%, respectively).

#### Site-dependence of hurricane damages

We did not found a significant site-effect on hurricane damages using the field campaign data (ANOVA’s Date-Site effects; Table 2; Fig. 5e). Before Irma, sites #2, #3, #4 had the same proportions of alive, decaying and dead trees (Fig. 5a) whereas less decaying and dead trees were found in sites #5, #6 and #7. After Irma, no living trees were found in the two subplots of site #1 (Fig. 5e). The absence of a significant site-effect after Irma was not caused by identical forest structure characteristics. Indeed, species and study sites were dependent before and after Irma (Fisher’s exact test, p-value < 0.0001). Moreover, means of trunk diameters and among study sites were not identical, as well as means of tree heights, either before or after Irma (Fig. 5b,c,f,g; Kruskal-Wallis rank sum test for all parameters, p-value < 0.0001).

### Results from remote sensing observations

**Figure 6:**
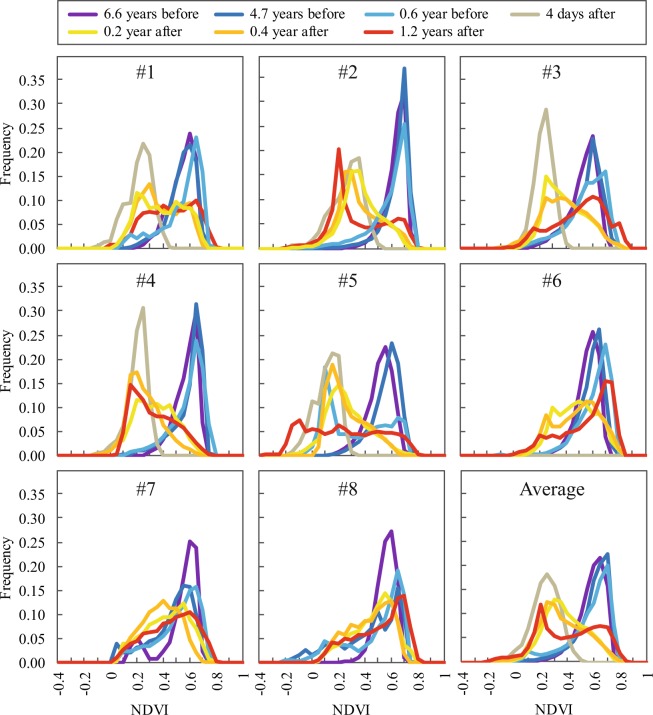
Distribution of NDVI values among study sites and at the island scale. Values greater than 0.40 correspond to healthy vegetation with high productivity, whereas values ranging from 0.1 to 0.4 generally correspond to bare ground, dead wood or vegetation with low productivity. Values less than 0 correspond to water.

In accordance with field campaigns, satellite observations revealed that the hurricane severely affected mangrove stands. Before Irma (−6.6 yr., −4.7 yr. and −0.6 yr.), the mean frequency distributions of NDVI values were very similar and showed that mangroves were in good health (Fig. 6; blue colours). NDVI values averaged 0.60 ± 0.14, and 91 ± 5% of values were higher than 0.40. Modes of the frequency distributions were centred on 0.60 ± 0.20 and corresponded to vigorous, productive mangrove vegetation with closed canopies. The distribution showed lower densities (9 ± 4%) between 0.10 and 0.40 which generally corresponded to vegetation with lower productivity, or with lower densities mixed with bare ground or water surfaces (see Methods section). Sites #7 and #8 showed a decrease of their dominant mode between 6.6 yr. and 4.8 yr. before Irma (Figs. 6, S1).

Immediately after Irma (+4 d.), the mean in NDVI values abruptly decreased to 0.36 ± 0.18 (Fig. 6). Dominant modes of frequency distributions clearly dropped down to values corresponding to the reflectance of low productivity vegetation, low densities vegetation mixed with bare ground, dead wood or defoliated trees. Only 9% of NDVI values corresponded to healthy canopies (NDVI >0.40), 81% corresponded to non-healthy vegetation or bare ground (NDVI >0.10 and <0.40), 10% corresponded to mix water and bare ground surfaces (NDVI <0.10).

The time series of NDVI values revealed that after Irma, the mangrove vegetation greenness rapidly recovered from the disturbance. As early as three months after Irma (+0.2 yr.), approximatively 48% of NDVI values increased to values of healthy canopies (Fig. S1). Fourteen months after Irma (+1.2 yr.), approximatively 54% of NDVI values corresponded to healthy canopies and 39% to vegetation with lower productivity, or with lower densities mixed with bare ground or water surfaces. The bimodal distribution of NDVI values 1.2 yr. after Irma showed that only part of the mangrove area recovered (Fig. 6). The proportion of pixels of high biomass production (i.e. NDVI >0.40) was significantly more variable across sites one year after Irma than it was the case seven years before (Fig. S1; Paired Levene test; p-value = 0.021), highlighting the existence of different rates of recovery between sites.

**Figure 7:**
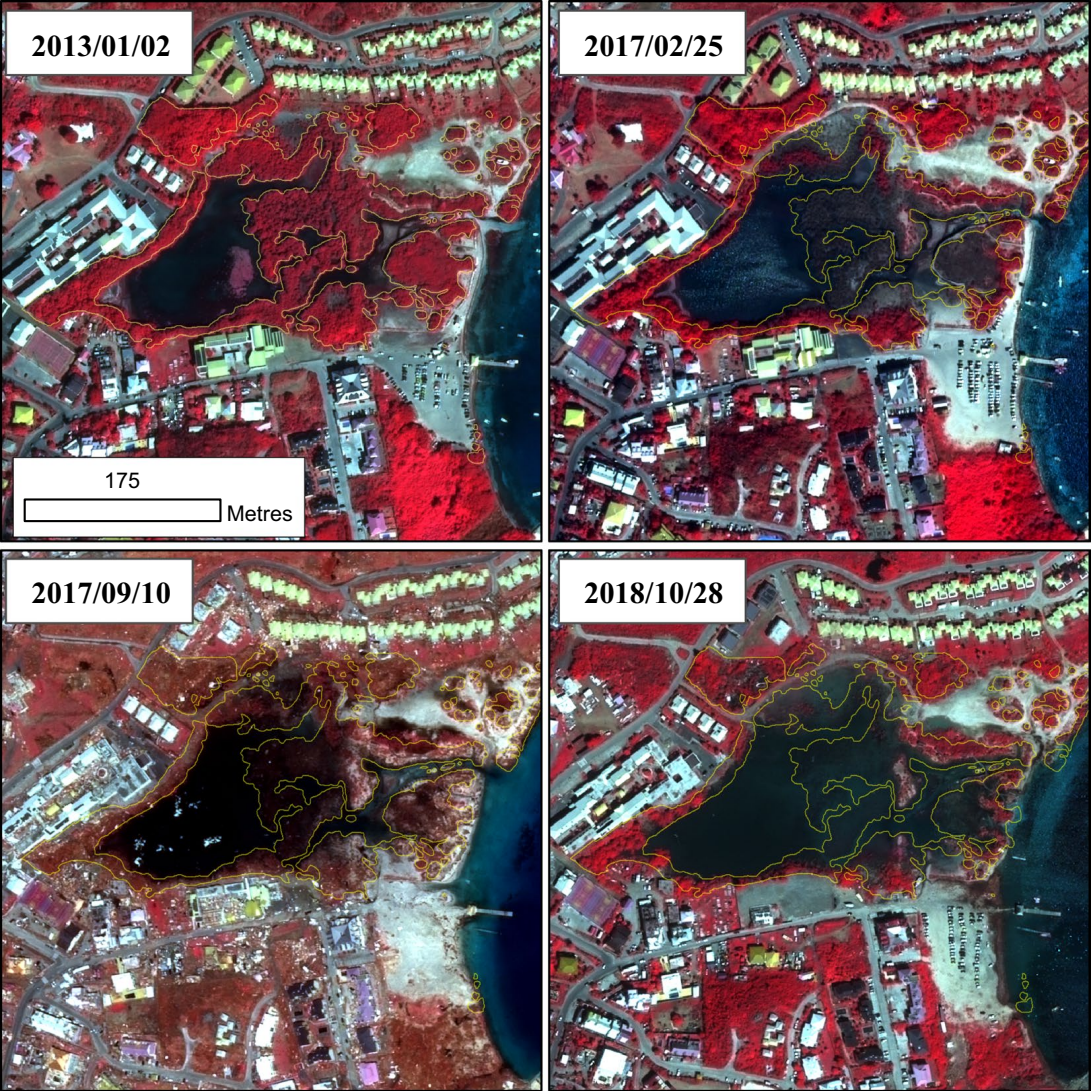
Absence of mangrove forest recovery in site #5 observed with very high resolution satellite images. The mangrove limit mapped on the field in 2011 is displayed in a thin yellow line. In 2013, the mangrove canopy was vigorous. About seven months before Irma (2017/02/25) a large portion of the mangrove cover was dying due to multiple human-induced stress factors, in particular obstruction of the water flow. About 14 months after Irma (2018/10/28), whilst most of the mangrove vegetation early recovered over the island, a large portion of mangroves in site #5 disappeared, replaced by water surface. The map in this figure was generated by ArcGIS 10.5 software package (ESRI, Redlands, CA, USA, http://resources.arcgis.com/en/home/). Contains information © 2013, 2017 & 2018, Distribution Airbus Defense & GEOSUD, France, all rights reserved. Commercial use prohibited.

Our analyses exhibited notable variations in recovery patterns among study sites (Fig. S1). Site #6 was the first to recover from the disturbance, followed by sites #8, #7, #3 and 1#. By contrast, sites #2, #4 and 5# poorly recovered. In particular, fourteen months after Irma, the major part of site #5 was unable to recover, with a first quartile at 0.02 and a mean at 0.27 ± 0.28 (Figs. 6, S1). Observation of satellite views exhibited that a very large portion of the mangrove situated in the middle of the pond disappeared, replaced by water surfaces (Fig. 6; #5 with NDVI <0; Fig. 7). Retrospective analysis of NDVI frequency distribution of site #5 revealed that mangroves exhibited a clear drop down in NDVI values prior to the hurricane. Indeed, the bimodal distribution of NDVI values were detected seven months before Irma (−0.6 yr.). Just before Irma, approximately 47% of NDVI values corresponded to non-healthy vegetation or bare ground (NDVI >0.10 and <0.40).

## Discussion

### Effects of site, species, trunk diameters and tree heights

Several studies reported that position of sites, species and morphological characteristics of trees modulate the damages in forests caused by cyclones^17–19^. Here, field inventories did not show significant site-effects nor effects of forest structure characteristics. Perhaps the huge force applied by Irma to the vegetation had overlap potential bio-mechanic and site differences. Moreover, the limited height of Saint Martin mangrove stands could have precluded bio-mechanic interactions. At the island scale however, satellite observations showed differences between sites immediately after the hurricane. Study sites situated on the east side of Saint Martin (facing the hurricane displacement, i.e. sites #1 to #5) were more impacted by canopy dieback than sites situated on the west side (i.e. site #6 to #8; Fig. 6). The origin of this pattern can be certainly attributed to wind directions and shadowing effects.

We found a species effect on hurricane damages. In the literature, the effect of species on hurricane impacts is controversial^20^. By instance, there is no agreement about the resistance capacity of *A. germinans* compared to *R. mangle*^18,21–26^. Here, field data showed that before Irma, species and tree health states were not related. *A.germinans* and *L. racemosa* were equally affected by decay and mortality. But after Irma, the species and tree health states relation were significant, suggesting a species-effect of hurricane damages. *C. erectus* and *L. racemosa* were less impacted by the hurricane than *A. germinans* and *R. mangle*.

Relationship between health states and trunk diameters or tree heights is also often unclear^7,20–27^. Taller stands are in theory the most damaged after cyclone landfalls^17,20,22,27–31^. Eppinga and colleagues^7^ found that neither individual stem size nor community size distributions mediated the Saba and Saint Eustatius forest’s response to hurricanes Irma and Maria. Here, our data showed that trunk diameters were independent of tree health states. The relationship with tree heights was more unclear. After Irma, tree health states were independent of tree heights (Fig. 4f), but it is difficult to know whether the hurricane broke all size of trees nor small trees better resisted.

### Recovery or mortality after cyclones

Relevant Supplementary Information has been provided by satellite observations. The assessment of hurricane impacts on mangrove forests using satellite observations has been widely conducted in the past^6,32–34^. Vegetation indices and in particular the NDVI, has been extensively used to detect the canopy cover dieback and recovery consecutive to cyclones^29,35–39^.

Here, NDVI showed massive diebacks at the island scale immediately after Irma and early recovery in the 2 following months. Long *et al*.^39^ showed that 18 months were necessary to detect resilience. Imbert (2018)^20^ showed that 23 years after Hugo in Guadeloupe (Lesser Antilles), a category 4 hurricane, fringe and scrub mangrove stands had not fully recovered unlike basin stands which was the most severely damaged. Early recovery detected on satellite scenes using NDVI is different of a full recovery of stand structure characteristics as reported by Imbert^20^. NDVI changes after cyclones mainly correspond to defoliation and re-foliation processes and not to the recovery of stand structures characteristics (DBH, tree heights, species composition, etc.).

In our study, the mangrove forest recovery can be attributed to the ability of *A. germinans* and *L. racemosa* to resprout from remaining plant material, unlike *R. mangle*. Recovery can also be attributed to the growth of juveniles in the forest understory^40^. *A. germinans* and *L. racemosa* are often more competitive for growing under open canopies than *R. mangle*. Asbridge and colleagues^6^ for instance, showed no or very limited mangrove recovery after a category 5 cyclone in a protected area of the North East Australia. Authors pointed out the persistent inundation that prevented seedling from growing and the inability *R. stylosa* species to resprout from remaining plant material. Some studies also showed that even if mangroves were able to early recover, the hydro-morphological changes induced by cyclones had prevented seedlings from growing, sometime with delayed mortality^41^.

Human-induced hydro-morphological changes prior to cyclones can also preclude the mangrove recovery after disturbances. Here, analyses showed a site-effect on canopy damages and recovery. Sites situated on the west side of the island were less impacted than those of the east side, and canopy recovery appeared to be more efficient. Observations revealed that site #5 was unable to recover and mortality increased with time from the disturbance (Figs. 6, 7 a nd S1). As opposed to the other study sites, site #5 was affected by vegetation decay seven months before the hurricane. The causes of such mortality need to be further investigated with the hypothesis of the synergistic effect of multiple human-induced stressors. During previous field campaigns carried out in 2015–2016^42^, alarming degradation rates were observed in several mangroves of the island. Embankments next to mangroves, illegal effluent of sewages and deposits of domestic wastes were widely observed, and in particular next site #5. Our data showed that approximately 50% of the mangrove area in site #5 was affected by mangrove decay 7 months before Irma. Mangrove dieback prior to Irma was due to an alteration of the flow regime due to urbanisation (especially parking development), probably combined with an intense dry season in 2015 and nutrient over-enrichment. The synergistic effect of multiple human-stressors combined with extreme events like erosion and drought has been highlighted in previous studies^5^. Here, mangroves of site #5 will certainly be unable to naturally recover from the disturbance. We suggest here that together with the situation of mangroves relative to the hurricane trajectory, the pressures exerted by over-urbanisation is a critical factor controlling the resistance and resilience of mangroves to hurricanes.

## Conclusion

The impact of hurricanes to mangroves is often difficult to assess due to the lack of comparable data between before and after the disturbance. Here, we have presented our data and analysis for the case of the island of Saint Martin, which faced hurricane Irma, graded H5 on the 5–6 September 2017. Field observations showed a significant species-effect in hurricane damages. Satellite observations showed a windward versus a leeward effect. Mangroves which have faced the hurricane trajectory on the east side of the island were more damaged. The early recovery of the mangrove canopy greenness has been widely observed demonstrating the high resilience ability of mangroves. However, analyses revealed that a mangrove area was unable to recover from the disturbance. We showed that this area was previously affected by forest dieback, due to several human-induced factors of alteration. The frequency of such very high energy hurricanes is predicted to increase in the coming century. Based on this study, we suggest that human-altered mangroves of this region will be weakened in the face of an intensification of such extreme events. We advocate to urgently preserve and restore human-degraded mangroves in order to guarantee all the valuable ecosystem services provided by this ecosystem.

## Methods

### Study area

Saint Martin is a 98 km² tropical island formed by sedimentary and volcanic rocks situated in the West Indies (Caribbean basin), approximately 300 km east of Puerto Rico and 200 km North-West of Guadeloupe (Fig. 1). The northern part of the island is attached to the French overseas collectivity of Saint Martin (Fig. 2a). The island has a tropical monsoon climate with a dry season from January to May and a wet season from June to December. The dry season is characterized by severe droughts which induce temporary water stress for mangroves. The small dimensions of the island and the low annual rainfall, allow only small freshwater resources, with few non-perennial rivers and limited groundwater^43^. The mean annual air temperature over the 1953–2015 period was 26.9 °C (data from Météo France website). The mean annual precipitation was 1 005 mm but varied annually depending on the number of passing hurricanes during the wet season. Tides are microtidal, mixed type, with a mean tidal range at spring tide of about 0.2 m.

The vegetation of Saint Martin is mainly xerophytic and grows on sedimentary and volcanic rocks. The coastal area is characterized by the presence of valleys ending with brackish waters in ponds sheltered behind sand bars and ancient coral reefs. Ponds are usually connected to the ocean by natural outlets but sedimentation can close connections with the sea. Mangroves are mainly found as fringing stands inside ponds (Fig. 2). Shrubby mangrove is the most common morphological type due to the water stress while wooded mangrove is rarer. True mangrove species in Saint Martin belong to the western floristic group^44^: *Rhizophora mangle* and *Avicennia germinans* dominate the outer mangrove fringe which is permanently inundated and where interstitial salinity ranges between 15–35 g/kg. Stands are mostly shrubby, multi-stemmed with a mean canopy height of 2–4 m. Landward, these species can occur in saltier areas as dwarf stands. *Laguncularia racemosa* can occur in association with *A. germinans* and *R. mangle*. Where salinity is low, *L. racemosa* can appear in monospecific old stands, 12–14 m height. *Conocarpus erectus* is ubiquitous over the coastal area. It can appear as dwarf stands (0.2–0.3 m) on ancient coral plateaus, shrubby stands (2–3 m) in back mangroves, and tree stands (10–12 m) on sand bars. Mangrove associates found in the periphery of Saint Martin’s mangroves are mainly *Ipomoea pes-caprae*, *Coccoloba uvifera*, *Sesuvium portulacastrum*. Since 1852 and within a 60 km radius, the Saint Martin Island has been struck by 74 storms from which 19 made landfall as hurricanes as defined by the Saffir-Simpson wind scale (Table S1). The mean return period for hurricanes H1 to H5 within a 60 km radius is 9.2 years.

### Description of hurricane Irma

Hurricane Irma started as a tropical depression west of Cape Verde Islands the 30^th^ August 2017 and ended in the continental United States (Missouri) on the 13^th^ September 2017^45^ (Fig. 1). According to the Saffir-Simpson hurricane wind scale, Irma was rated as a category 5 hurricane on the 05^th^ September 2017, 130 km east-southeast of Barbuda. It made landfall as a category 5 hurricane on the Saint Martin Island on the 5–6 September 2017 at night and high tide (0.61 m). It was the strongest observed hurricane for this island, with winds exceeding 287 km/h and waves reaching 8 m^45^. Tide gauges from Antigua and Barbuda recorded a peak water level of 2.4 m above the mean higher high water, suggesting that inundation occurred far across the shore. This inordinately powerful hurricane damaged about 90% of housing structures, displaced about 7000 people and caused 11 deaths in Saint Martin and Saint Barthelemy islands. Winds were highly damaging and the combined effect of winds, waves, water suspended sediment generated destructive storm surges at the coast.

### Data from field campaigns

In June 2011, a first field survey was conducted in order to map mangrove extent in the French part of the island. A differential global positioning system GeoXH 6000 (Trimble, Sunnyvale, CA, USA) was used in the field to control the mapping based on a satellite scene. Pre-disturbance forest structure inventories were conducted in sites *#2, #3, #4, #5, #6* and *#7* (Fig. 2). Site pre-disturbance inventories consisted in several 5 m by 5 m square plots where trees were counted, species identified, tree height and trunk diameter at breast height (DBH in cm) were measured using a tape and a telescopic pole. Tree vitality (alive, decaying, dead) was characterised. About 8 months after Irma (April 2018), post-hurricane forest structure inventories were conducted in sites #1, #2, #3 and #5 (Fig. 2). For site #2, #3 and #5, plots locations were different between 2011 and 2018 but were neighbours and represented the same stands (Table S2). The classification of tree vitality was different from the previous census also, as five classes were distinguished in order to more precisely characterise the hurricane impact on vegetation (Table 3). For the comparison purpose, correspondences were found between pre- and post-disturbance vitality classes.

**Table 3:** Definition and correspondence of vitality classes in the pre- (2011) and post-disturbance (2018) forest inventories.

Class in 2011	Class in 2018	Description
Dead	Dead	No leaves, dry wood.
Decaying	Seriously decaying	Defoliated, uprooted, broken, with a least 1 branch alive with some leaves.
	Decaying	Defoliated with a least 1 branch alive with some leaves.
Alive	Good vigour	2/3 of the crown vigorous
	Very good vigour	Crown totally vigorous

### Data from aerial and satellite images

We used the archives of aerial and satellite images listed in Table 1 in order to map the mangrove extent and to assess the vegetation vitality before and after Irma. Six orthorectified, pansharpened (pixel size = 0.5 * 0.5 m) Pléiades satellite images acquired between 2013 and 2018 were retrieved at https://www.intelligence-airbusds.com/geostore/. They consisted in 4 spectral bands (blue, green, red and near-infrared) stacked into a single image. In addition, the field campaign preceding Irma in 2011 had required a multispectral WorldView 2 satellite image acquired the 26 February 2011 (https://www.digitalglobe.com/). This dataset consisted in 4 spectral bands (blue, green, red and near-infrared) stacked into a single multiband image with at 2 m by 2 m pixel resolution, and in a single band panchromatic image at 0.5 m by 0.5 m resolution. We conducted image fusion using a simple mean algorithm to obtain a single 4 channels image with the finest pixel resolution (0.5 m by 0.5 m). To precisely control all images alignment, we used orthorectified digital aerial images acquired in 2004 as targets. These images were provided by the French National Institute of Geographic and Forest Information (IGN; http://professionnels.ign.fr/orthoimages). All the satellite scenes listed in Table 1 were split into smaller parts corresponding to the studied sites and then geometrically corrected separately, based on target rasters for identifying ground control points. This procedure was used to ensure the best accuracy in image overlay. All analyses were conducted using the ArcGis software package (ESRI, Redlands, CA, USA).

### Mapping of the mangrove extent in 2011

The 2011 WordView 2 satellite scene was used to map the extent of mangroves by photo-interpretation and manual digitalisation on the computer screen. During the field survey in 2011, the mangrove map was uploaded to the DGPS Trimble GEO XH 6000 and annotated on the field for further correction at the laboratory. Only true species of mangroves, i.e. *A. germinans*, *L. racemosa*, *R. mangle*. and *C. erectus* were considered as a whole unit for the mangrove extent.

### Time series of vegetation index

The normalized difference vegetation index^46^ (NDVI) was calculated on satellite images for detecting mangrove vegetation vitality (greenness). Chlorophyll causes considerable absorption of incident red light, whereas the spongy mesophyll leaf structure produces considerable reflectance in the near-infrared region of the spectrum. *NDVI* values range from −1.0 to +1.0. Surface waters tend to negative values. Areas of bare soil, rock and sand generally show very low positive NDVI values (for example, +0.1 or less). Sparse or poorly productive vegetation such as shrubs usually result in moderate NDVI values (approximately +0.1 to +0.3). High NDVI values (approximately +0.6 to +1.0) correspond to dense vegetation with high levels of productivity (growing healthy vegetation). The NDVI threshold value which separated poorly and high productive vegetation was chosen to be 0.4 in Fig. S1. Calculations were only carried out within the 2011 mangrove extent validated by field survey (see section 3.5). NDVI was calculated on every satellite scene.

### Statistical analyses of forest inventories

Association between qualitative variables were tested using the Pearson’s Chi-square test on contingency tables. When sample sizes were lower than 5 we used the Fisher’s exact test instead. These tests were used to investigate whether tree health states, species and study sites were dependent to each other, and to trunk diameters and tree heights classed into several size categories. Categories of trunk diameters were: Very thin 0–2 cm; Thin = 2–5 cm; Medium = 5–10 cm; Large = 10–20 cm; Very large >20 cm. Categories of tree heights were: Very low = 0–1.5 m; Low = 1.5–3 m; Medium = 3–5 m; High >5 m. All the analyses were conducted twice, in 2011 and in 2018 (i.e. before and after Irma).

Comparisons between means of quantitative variables among control variables (study sites, inventory date) were carried out with Kruskal-Wallis rank sum test.

ANOVAs were used to investigate differences of tree density, diameter, height and health among dates, sites and tree species (Table 2). We limited our analysis to *A. germinans* and *L. racemosa* species, being the only species with inventory data available at both dates. We considered the “Date Site” and the “Date Species” interaction terms in the ANOVAs in order to investigate possible differences of Irma’s effect between sites and between species. Raw data of the forest inventories were aggregated at the plot level prior to this analysis (number of trees per unit area, mean diameter, mean height, and proportion of living trees in each plot). Residuals were checked for normality and homoscedasticity using a combination of Shapiro-Wilk and Levene’s test.

All statistical analyses were conducted using XLSTAT software package (Addinsoft, Paris, France). A *p*-value less than or equal to 0.05 was considered as significant.

### Analyses of vegetation indices

We used 104 028 data points (1 point per pixel) within the 2011 mangrove cover extent to analyse NDVI values. The number of data points differed among study sites (#1 = 1 253; #2 = 46 043; #2 = 24 089; #4 = 6 141; #5 = 8 707; #6 = 12 441; #7 = 3 607; #8 = 1 748).

The distributions of NDVI values among the 6 dates and 8 sites were represented as histograms, using a 0.05 bin width. The distributions of NDVI values at the island level was calculated in a similar manner, by using all the 104 028 pixels, thus approximating the probability density function of NDVI at the sampled pixels.

## Supplementary information

Suplementary Information

Dataset 1

Dataset 2

## Data Availability

Raw data used in this study are available online as Supplementary Dataset.

## References

[1] Worthington, T. & Spalding, M. Mangrove restoration potential: a global map highlighting a critical opportunity. 1–19 (UICN, 2018).

[2] Lee SY (2014). Ecological role and services of tropical mangrove ecosystems: a reassessment. Glob. Ecol. Biogeogr..

[3] Murdiyarso D (2015). The potential of Indonesian mangrove forests for global climate change mitigation. Nat. Clim. Chang..

[4] Carugati L (2018). Impact of mangrove forests degradation on biodiversity and ecosystem functioning. Sci. Rep..

[5] Sippo JZ, Lovelock CE, Santos IR, Sanders CJ, Maher DT (2018). Mangrove mortality in a changing climate: An overview. Estuarine, Coastal and Shelf Science.

[6] Asbridge E, Lucas R, Rogers K, Accad A (2018). The extent of mangrove change and potential for recovery following severe tropical cyclone Yasi, Hinchinbrook Island, Queensland, Australia. Ecol. Evol..

[7] Eppinga MB, Pucko CA (2018). The impact of hurricanes Irma and Maria on the forest ecosystems of Saba and St. Eustatius, northern Caribbean. Biotropica.

[8] Roth LC (1992). Hurricanes and mangrove regeneration: effects of hurricane Joan, October 1988, on the vegetation of Isla del Venado, Bluefields, Nicaragua. Biotropica.

[9] Simard M (2019). Mangrove canopy height globally related to precipitation, temperature and cyclone frequency. Nat. Geosci..

[10] O’Leary JK (2017). The resilience of marine ecosystems to climatic disturbances. BioScience.

[11] Alongi DM (2008). Mangrove forests: resilience, protection from tsunamis, and responses to global climate change. Estuar. Coast. Shelf Sci..

[12] Feller IC (2015). Nutrient enrichment intensifies hurricane impact in scrub mangrove ecosystems in the Indian River Lagoon, Florida, USA. Ecology.

[13] Bender MA (2010). Modeled impact of anthropogenic warming on the frequency of intense Atlantic hurricanes. Science.

[14] IPCC. Working Group I Contribution to the IPCC Fifth Assessment Report, Climate Change 2013: The Physical Science Basis. Working Group I Contribituion to the IPCC Fifth Assessment Report, Climate Change 2013: The Physical Science Basis (2013).

[15] Burn MJ, Palmer SE (2015). Atlantic hurricane activity during the last millennium. Sci. Rep..

[16] Knutson TR (2015). Global projections of intense tropical cyclone activity for the late twenty-first century from dynamical downscaling of CMIP5/RCP4.5 scenarios. J. Clim..

[17] Doyle TW, Smith TJ, Robblee MB (1992). Wind damage effects of Hurricane Andrew on mangrove communities along the southwest coast of Florida, USA. J. Coast. Res..

[18] Milbrandt EC, Greenawalt-Boswell JM, Sokoloff PD, Bortone SA (2006). Impact and response of southwest Florida mangroves to the 2004 hurricane season. Estuaries and Coasts.

[19] Doyle TW, Krauss KW, Wells CJ (2009). Landscape analysis and pattern of hurricane impact and circulation on mangrove forests of the Everglades. Wetlands.

[20] Imbert D (2018). Hurricane disturbance and forest dynamics in east Caribbean mangroves. Ecosphere.

[21] Smith TJ, Robblee MB, Wanless HR, Doyle TW (1994). Mangroves, hurricanes, and lightning strikes. Bioscience.

[22] Imbert D, Labbé P, Rousteau A (1996). Hurricane damage and forest structure in Guadeloupe, French West Indies. J. Trop. Ecol..

[23] Kovacs JM, Blanco-Correa M, Flores-Verdugo F (2001). A Logistic Regression Model of Hurricane Impacts in a Mangrove Forest of the Mexican Pacific. J. Coast. Res..

[24] Shearman P, Bryan J, Walsh JP (2013). Trends in Deltaic Change over Three Decades in the Asia-Pacific Region. J. Coast. Res..

[25] Ross MS (2006). Early post-hurricane stand development in Fringe mangrove forests of contrasting productivity. Plant Ecol..

[26] Smith Thomas J., Anderson Gordon H., Balentine Karen, Tiling Ginger, Ward Greg A., Whelan Kevin R. T. (2009). Cumulative impacts of hurricanes on Florida mangrove ecosystems: Sediment deposition, storm surges and vegetation. Wetlands.

[27] Sherman RE, Fahey TJ (2001). Hurricane impacts on a mangrove forest in the Dominican Republic: damage patterns and early recovery. Biotropica.

[28] Walker LR (2006). Tree Damage and recovery from hurricane Hugo in Luquillo xxperimental forest, Puerto Rico. Biotropica.

[29] Vandecar KL (2011). High mortality for rare species following hurricane disturbance in the southern Yucatán. Biotropica.

[30] Lewis RJ, Bannar-Martin KH (2012). The impact of cyclone Fanele on a tropical dry forest in Madagascar. Biotropica.

[31] Tanner EVJ, Rodriguez-Sanchez F, Healey JR, Holdaway RJ, Bellingham PJ (2014). Long-term hurricane damage effects on tropical forest tree growth and mortality. Ecology.

[32] Giri C (2008). Mangrove forest distributions and dynamics (19752005) of the tsunami-affected region of Asia. J. Biogeogr..

[33] Ayala-Silva T, Twumasi YA (2004). Hurricane Georges and vegetation change in Puerto Rico using AVHRR satellite data. Int. J. Remote Sens..

[34] Aosier, B., Kaneko, M. & Takada, M. Evaluation of the forest damage by typhoon using remote sensing technique. In *International Geoscience and Remote Sensing Symposium (IGARSS)* (2007).

[35] Rodgers JC, Murrah AW, Cooke WH (2009). The impact of hurricane katrina on the coastal vegetation of the weeks bay reserve, alabama from NDVI data. Estuaries and Coasts.

[36] Kamthonkiat D, Rodfai C, Saiwanrungkul A, Koshimura S, Matsuoka M (2011). Geoinformatics in mangrove monitoring: Damage and recovery after the 2004 Indian Ocean tsunami in Phang Nga, Thailand. Nat. Hazards Earth Syst. Sci..

[37] Macamo CCF, Massuanganhe E, Nicolau DK, Bandeira SO, Adams JB (2016). Mangrove’s response to cyclone Eline (2000): What is happening 14 years later. Aquat. Bot..

[38] Han X, Feng L, Hu C, Kramer P (2018). Hurricane-induced changes in the Everglades National Park mangrove forest: Landsat observations between 1985 and 2017. *J. Geophys. Res*. Biogeosciences.

[39] Long J, Giri C, Primavera J, Trivedi M (2016). Damage and recovery assessment of the Philippines’ mangroves following Super Typhoon Haiyan. Mar. Pollut. Bull..

[40] Baldwin A, Egnotovich M, Ford M, Platt W (2001). Regeneration in fringe mangrove forests damaged by Hurricane Andrew. Plant Ecology.

[41] Radabaugh, K. R. *et al*. Mangrove damage, delayed mortality, and early recovery following hurricane Irma at two landfall sites in southwest Florida, USA. *Estuaries and Coasts*., 10.1007/s12237-019-00564-8 (2019).

[42] Herteman, M. Accompagnement des gestionnaires au suivi des étangs et expertise des mangroves de Saint Martin. 1–24 (Ecosphere, 2016).

[43] Lambs L, Bompy F, Dulormne M (2018). Using an “isotopic spike” from tropical storm to understand water exchange on large scale: case study of Hurricane Rafael in the Lesser Antilles archipelago, October 2012. *Rapid Comm. In*. Mass Spectrom.

[44] Tomlinson, P. B. *The botany of mangroves* (1986).

[45] Cangialosi, J. P., Latto, A. S. & Berg, R. *National Hurricane Center Tropical Cyclone Report. Hurricane Irma*. at, http://base-documentaire.pole-zh-outremer.org/documents/Docs_lies/2017/04/25/A1493149777SD_Rapport_Mang_Accompagnement_Expertise_etangs_SXM_avril2017.pdf (ATEN, CELRL, 2018).

[46] Tucker CJ (1979). Red and photographic infrared linear combinations for monitoring vegetation. Remote Sens. Environ..

