# Supplementary material for: Damages caused by hurricane Irma in the human-degraded mangroves of Saint Martin (Caribbean)
Source: Sci Rep. 2019 Dec 12;9:18971. doi: 10.1038/s41598-019-55393-3 (PMC6908650; doi:10.1038/s41598-019-55393-3)
Supplement: Supplementary file 1 — Suplementary Information [file 41598_2019_55393_MOESM1_ESM.docx]

Damages caused by hurricane Irma in the human-degraded mangroves of Saint Martin (Caribbean)

Walcker, R.*^1^, Laplanche, C.^1^, Herteman, M.^2^, Lambs, L. ^1^, Fromard, F.^1^

***Laboratory of origin***:

^1^ EcoLab, Université de Toulouse, CNRS, Toulouse, France.

^2^ Nature & Développement, Martinique, France.

****Corresponding author:***

Romain Walcker: [romain.walcker@univ-tlse3.fr](mailto:romain.walcker@univ-tlse3.fr)

**List of supplementary information**

**Supplementary Figure S1.** Fig. S1 Variability in rates of canopy greenness recovery.

**Supplementary Table S1.** Table S1 List of hurricanes that passed within a 60 km circular area and ranked more than category 1 (H1) on the Saffir-Simpson wind scale. Data from <https://coast.noaa.gov/hurricanes/>.

**Supplementary Table S2.** Table S2 Summary of inventoried forest structure parameters before (June 2011, 266 trees) and after Irma (April 2018, 169 trees).


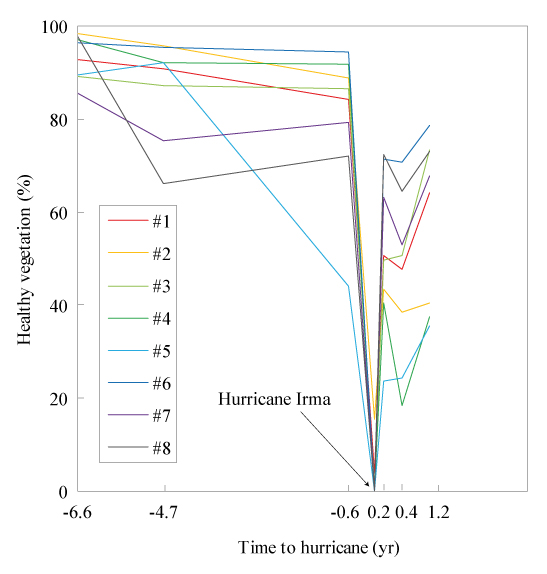


**Fig. S1** Variability in rates of canopy greenness recovery. Healthy is defined here as NDVI values >0.40.

**Table S1** List of hurricanes that passed within a 60 km circular area and ranked more than category 1 (H1) on the Saffir-Simpson wind scale. Data from <https://coast.noaa.gov/hurricanes/>.

| *Name* | *Date on Saint Martin* | *Category* | *Wind speed* | *Origin* |
| --- | --- | --- | --- | --- |
| Irma | 6^th^ September 2017 | H5 | 287 km/h | South-East |
| Gonzalo | 13^th^ October 2014 | H1 | 139 km/h | South-East |
| Earl | 29^th^ August 2010 | H3 | 194 km/h | South-East |
| Debby | 22^th^ August 2000 | H1 | 120 km/h | South-East |
| Lenny | 19^th^ November 1999 | H2 | 250 km/h | South-East |
| Jose | 21^st^ October 1999 | H1 | 139 km/h | South-East |
| Bertha | 08^th^ July 1996 | H1 | 130 km/h | South-East |
| Luis | 06^th^ September 1995 | H4 | 213 km/h | South-East |
| Donna | 05^th^ September 1960 | H3 | 203 km/h | South-East |
| Alice | 02^nd^ January 1955 | H1 | 111 km/h | North-East |
| Dog | 01^st^ September 1950 | H3 | 194 km/h | South-East |
| Unnamed | 26^th^ September 1932 | H4 | 222 km/h | South-East |
| Unnamed | 16^th^ September 1922 | H3 | 185 km/h | South-East |
| Unnamed | 02^nd^ September 1906 | H2 | 166 km/h | South-East |
| Unnamed | 09^th^ September 1899 | H3 | 185 km/h | South-East |
| Unnamed | 12^th^ September 1898 | H2 | 175 km/h | South |
| Unnamed | 12^th^ September 1876 | H2 | 166 km/h | East |
| Unnamed | 21^st^ August 1871 | H3 | 185 km/h | South-East |
| Unnamed | 23^th^ September 1852 | H1 | 129 km/h | South-East |

Table S2 Summary of inventoried forest structure parameters before (June 2011, 266 trees) and after Irma (April 2018, 169 trees).

| *Site* | *Plot* | *Date* | *Size* | *Lat** | *Lon** | *Tree* | *Avi*** | *Rhi*** | *Lag*** | *Con*** | *Died* | *Decaying* | *Alive* | *DBH**** | *Height**** |
| --- | --- | --- | --- | --- | --- | --- | --- | --- | --- | --- | --- | --- | --- | --- | --- |
| *Id* | *Id* | *yyyy* | *m*m* | *dd* | *dd* | *count* | *count* | *count* | *count* | *count* | *count* | *count* | *count* | *cm* | *m* |
| #2 | 1 | 2011 | 5*5 | 18.075920 | -63.023468 | 30 | 0 | 1 | 29 | 0 | 6 | 7 | 17 | 2.4(0.9) | 4.7(0.7) |
| #3 | 1 | 2011 | 5*5 | 18.082011 | -63.017501 | 7 | 2 | 0 | 5 | 0 | 1 | 2 | 4 | 4.5(0.9) | 5.8(1.5) |
| #4 | 1 | 2011 | 5*5 | 18.096855 | -63.033297 | 9 | 5 | 0 | 4 | 0 | 1 | 3 | 5 | 15.8(2.7) | 6.4(1.9) |
| #5 | 1 | 2011 | 5*5 | 18.106611 | -63.027078 | 52 | 10 | 0 | 42 | 0 | 2 | 5 | 45 | 2.2(0.8) | 3.7(0.6) |
| #5 | 2 | 2011 | 5*5 | 18.106486 | -63.025878 | 17 | 5 | 0 | 12 | 0 | 4 | 7 | 6 | 5.2(0.9) | 2.3(0.5) |
| #5 | 3 | 2011 | 5*5 | 18.105930 | -63.026112 | 93 | 53 | 0 | 40 | 0 | 0 | 41 | 52 | 3.1(1.0) | 2.1(0.4) |
| #6 | 1 | 2011 | 5*5 | 18.099064 | -63.048720 | 15 | 3 | 0 | 12 | 0 | 0 | 0 | 15 | 2.7(10) | 2.3(0.5) |
| #7 | 1 | 2011 | 5*5 | 18.093588 | -63.074416 | 43 | 3 | 0 | 40 | 0 | 4 | 4 | 35 | 4.3(1.1) | 4.9(1.5) |
| - | - | - | - | - | - | 266 | 81 | 1 | 184 | 0 | 18 | 69 | 179 | 3.6(2.7) | 3.4 (1.6) |
| #1 | 1 | 2018 | 10*10 | 18.062777 | -63.015277 | 7 | 7 | 0 | 0 | 0 | 3 | 4 | 0 | 1.9(0.4) | 2.1(0.4) |
| #1 | 2 | 2018 | 10*10 | 18.062773 | -63.014437 | 11 | 2 | 0 | 8 | 1 | 4 | 7 | 0 | 2.4(0.6) | 2.2(0.6) |
| #2 | 1 | 2018 | 10*10 | 18.111624 | -63.019462 | 15 | 1 | 1 | 13 | 0 | 8 | 5 | 2 | 3.4(3.0) | 2.1(0.7) |
| #2 | 2 | 2018 | 10*10 | 18.100003 | -63.016952 | 17 | 1 | 12 | 4 | 0 | 7 | 8 | 2 | 9.2(2.8) | 2.2(0.6) |
| #2 | 3 | 2018 | 10*10 | 18.116377 | -63.018922 | 15 | 0 | 15 | 0 | 0 | 13 | 2 | 0 | 17.5(8.8) | 3.3(1.6) |
| #3 | 1 | 2018 | 10*10 | 18.082220 | -63.019148 | 9 | 7 | 0 | 0 | 2 | 6 | 2 | 1 | 12.9(7.4) | 2.9(1.9) |
| #3 | 2 | 2018 | 10*10 | 18.078590 | -63.017767 | 12 | 0 | 0 | 10 | 2 | 0 | 11 | 1 | 9.4(7.4) | 5.2(2.1) |
| #3 | 3 | 2018 | 10*10 | 18.082765 | -63.020543 | 12 | 0 | 3 | 9 | 0 | 3 | 6 | 3 | 16.7(11.7) | 3.8(1.9) |
| #5 | 1 | 2018 | 10*10 | 18.106390 | -63.027225 | 12 | 11 | 0 | 0 | 1 | 8 | 4 | 0 | 16.0(4.1) | 2.1(0.4) |
| #5 | 2 | 2018 | 10*10 | 18.106387 | -63.026666 | 15 | 13 | 2 | 0 | 0 | 9 | 6 | 0 | 17.6(4.1) | 1.9(0.7) |
| #5 | 3 | 2018 | 10*10 | 18.106668 | -63.025834 | 15 | 1 | 0 | 12 | 2 | 1 | 10 | 4 | 9.9(2.0) | 1.5(0.6) |
| #5 | 4 | 2018 | 10*10 | 18.105834 | -63.026666 | 15 | 4 | 1 | 10 | 0 | 15 | 0 | 0 | 10.0(2.2) | 1.5(0.6) |
| #5 | 5 | 2018 | 10*10 | 18.105819 | -63.029718 | 14 | 4 | 0 | 9 | 1 | 0 | 9 | 5 | 9.9(2.8) | 1.9(0.6) |
| - | - | - | - | - | - | 169 | 51 | 34 | 75 | 9 | 77 | 74 | 18 | 10.8(7.3) | 2.5(1.5) |

* Geographic coordinates are given onto the WGS84 ellipsoid. ** *Avi* = *Avicennia germinans*; *Rhi* = *Rhizophora mangle*; *Lag* = *Laguncularia racemosa* ; *Con* = *Conocarpus erectus*. ***The plot arithmetic mean is given with its standard deviation in parenthesis
